# 3-D-Druck in der Chirurgie: Relevanz der Bewertung der Technologiereife in Forschungsstudien zum Bioprinting

**DOI:** 10.1007/s00104-024-02197-5

**Published:** 2024-12-04

**Authors:** Markus Laubach, Hanna Hartmann, Boris M. Holzapfel, Susanne Mayer-Wagner, Katja Schenke-Layland, Dietmar W. Hutmacher

**Affiliations:** 1 Klinik für Orthopädie und Unfallchirurgie, Muskuloskelettales Universitätszentrum München (MUM), LMU Klinikum, LMU München, Marchioninistr. 15, 81377 München, Deutschland; 2 NMI Naturwissenschaftliches und Medizinisches Institut an der Universität Tübingen, Markwiesenstr. 55, 72770 Reutlingen, Deutschland; 3 Institut für Biomedical Engineering, Abteilung für Medizintechnik und Regenerative Medizin, Eberhard Karls Universität Tübingen, Silcherstr. 7/1, 72076 Tübingen, Deutschland; 4 Max Planck Queensland Centre (MPQC) for the Materials Science of Extracellular Matrices, Queensland University of Technology, QLD 4000, Brisbane, Australien

**Keywords:** 3‑D-Druck, Bioprinting, Technologiereifegrad, Klinische Translation, Innovationsbewertung, 3D printing, Bioprinting, Technology readiness level, Clinical translation, Innovation assessment

## Abstract

Biologische 3‑D-Druckverfahren (sog. Bioprinting) sind eine Erweiterung dessen, was in den American Society for Testing and Materials(ASTM)- und International Organization for Standardization(ISO)-Normen als additive Fertigung definiert ist, und basieren auf dem automatisierten Druck von lebenden Zellen und Biomaterialien. Forschende und Expertinnen und Experten im Bereich der Biomaterialwissenschaften, der Gewebezüchtung und regenerativen Medizin („tissue engineering and regenerative medicine“, TE&RM) verweisen stets auf das Potenzial biologischer 3‑D-Druckverfahren und in Fachartikeln wird regelmäßig dessen baldige klinische Anwendung angekündigt. Wir argumentieren in dieser Arbeit, dass diese Ankündigungen regelhaft verfrüht und kontraproduktiv sind, da sie sich stark auf den technologischen Fortschritt konzentrieren, jedoch in der Regel die kritischen Phasen ignorieren, die durchlaufen werden müssen, um erfolgreich die Translation einer Technologie auf den Gesundheitsmarkt zu erzielen. Die Technologiereifegradskala („technology readiness level“, TRL) ist ein potenziell nützliches Instrument zur Messung der relativen Reife einer Technologie in Bezug auf die Überwindung einer Reihe kritischer Meilensteine. Wir schlagen eine Adaptierung der TRL-Skala vor und nutzen diese, um den aktuellen Stand der Forschung zu biologischen 3‑D-Druckverfahren zu diskutieren. Abschließend geben wir konkrete Empfehlungen zur Optimierung zukünftiger Forschungsprojekte, um den Weg für klinische Anwendungen des biologischen 3‑D-Drucks zu ebnen und damit einen direkten positiven Einfluss auf die chirurgische Patientenversorgung zu erzielen.

## Abstract (en)

Biological 3D printing (bioprinting) is an extension of what is defined as additive manufacturing in the American Society for Testing and Materials (ASTM) and International Organization for Standardization (ISO) standards and is based on the automated printing of living cells and biomaterials. Researchers and experts in the field of biomaterial science, tissue engineering and regenerative medicine (TE&RM) are constantly pointing to the potential of biological 3D printing and scientific articles regularly announce the imminent clinical application. We argue in this article that these announcements are often premature and counterproductive as they focus heavily on technological progress but regularly ignore the critical stages that need to be completed in order to successfully translate a technology into the healthcare market. The technology readiness level (TRL) scale is a potentially useful tool for measuring the relative maturity of a technology in terms of overcoming a series of critical milestones. We propose an adaptation of the TRL scale and use it to discuss the current state of research on biological 3D printing. Finally, we provide specific recommendations for optimizing future research projects to pave the way for clinical applications of biological 3D printing and thus achieve a direct positive impact on surgical patient care.

## Hintergrund

Der dreidimensionale Druck (3-D-Druck), auch bekannt als additive Fertigung oder „rapid prototyping“, wurde erstmals 1986 patentiert und umfasst heute eine Vielzahl unterschiedlicher Technologien, bei denen Material Schicht für Schicht aufgetragen, verbunden oder verfestigt wird, um aus einer digitalen Datei ein physisches Objekt zu erstellen. Das sog. „biologische 3‑D-Druckverfahren“ (sog. Bioprinting) ist eine Ausweitung der additiven Fertigungstechnologien auf biologische Systeme, die den automatisierten Druck von lebenden Zellen und Biomaterialien beinhaltet ([1, 2]; Infobox 1: Definition 1). In der Biomaterialienforschung und der regenerativen Medizin („tissue engineering and regenerative medicine“, TE&RM) werden Strategien des biologischen 3‑D-Druckverfahrens verstärkt als bevorzugtes Werkzeug zur Lösung komplexer und langwieriger Herausforderungen, einschließlich der Forschungsaufgabe der Nachbildung der internen Komplexität eines Gewebes und der Gefäßbildung, eingesetzt [3, 4]. Zudem wird in aktuellen Studien diskutiert, dass biologisch gedruckte Gewebe die Regeneration und Heilung möglicherweise unterstützen könnten, indem sie präzise an die individuellen Bedürfnisse des Patienten angepasst werden, was als potenzieller Fortschritt in der regenerativen Medizin und chirurgischen Praxis betrachtet wird [5]. In einer bibliometrischen Analyse aus dem Jahr 2021 wurde das biologische 3‑D-Druckverfahren als eines der wichtigsten Forschungsinteressen der TE&RM-Gemeinschaft in den letzten Jahren identifiziert [6]. Zu den potenziellen Anwendungen des biologischen 3‑D-Druckverfahrens zählen unter anderem: implantierbare 3‑D-gedruckte Konstrukte [1], In-vitro-Krankheitsmodelle [7] und Modelle für die Arzneimittelforschung und toxikologische Tests [8].

Auch wenn biologische 3‑D-Druckverfahren in diesen Bereichen relevante Rollen spielen, sind die von der Wissenschaft und der Industrie vorgeschlagenen Fristen bis zur Kommerzialisierung oft unrealistisch. In Fachartikeln sowie auf Unternehmenswebseiten und -broschüren wird das Aufkommen des biologischen 3‑D-Druckverfahrens und dessen Umsetzung in die klinische Anwendung häufig als unmittelbar bevorstehend skizziert [9]. Diese Ankündigungen erachten wir allerdings zumeist als verfrüht und kontraproduktiv; diese verharmlosen oder ignorieren die kritischen Phasen, die durchlaufen werden müssen, um eine Technologie vom Labor ans Klinikbett („from bench to bedside“) und/oder auf den (Gesundheits‑)Markt zu bringen. Unrealistische, oftmals zu optimistische Behauptungen über den Zeitplan oder die potenziellen Nutzen einer Technologie können zu überhöhten Erwartungen aller Interessengruppen beitragen, einschließlich der wissenschaftlichen und akademischen Gemeinschaft selbst, aber vor allem der Öffentlichkeit und der Investoren [10, 11]. Im Fall des biologischen 3‑D-Druckverfahrens nähren solche Behauptungen ein Narrativ in den populärwissenschaftlichen Medien, die diese Technologie als industrietauglicher darstellen, als diese womöglich in einzelnen Fällen tatsächlich ist. So wurde beispielsweise in einem Bericht aus dem Jahr 2019 über implantierbare Konstrukte hergestellt mit biologischen 3‑D-Druckverfahren dies in den Medien fälschlicherweise als Möglichkeit dargestellt, ein menschliches Herz in Originalgröße zu drucken [12]. Auch Vertreterinnen und Vertreter nationaler und internationaler Zulassungsbehörden haben sich offensichtlich von diesen Berichten täuschen lassen, denn einige Monate später veröffentlichte die International Coalition of Medicines Regulatory Authorities (ICMRA) eine Fallstudie zum biologischen 3‑D-Druckverfahren, in der sie das „erfolgreich gedruckte menschliche Herz“ ankündigte [13].

**Abb. 1:**
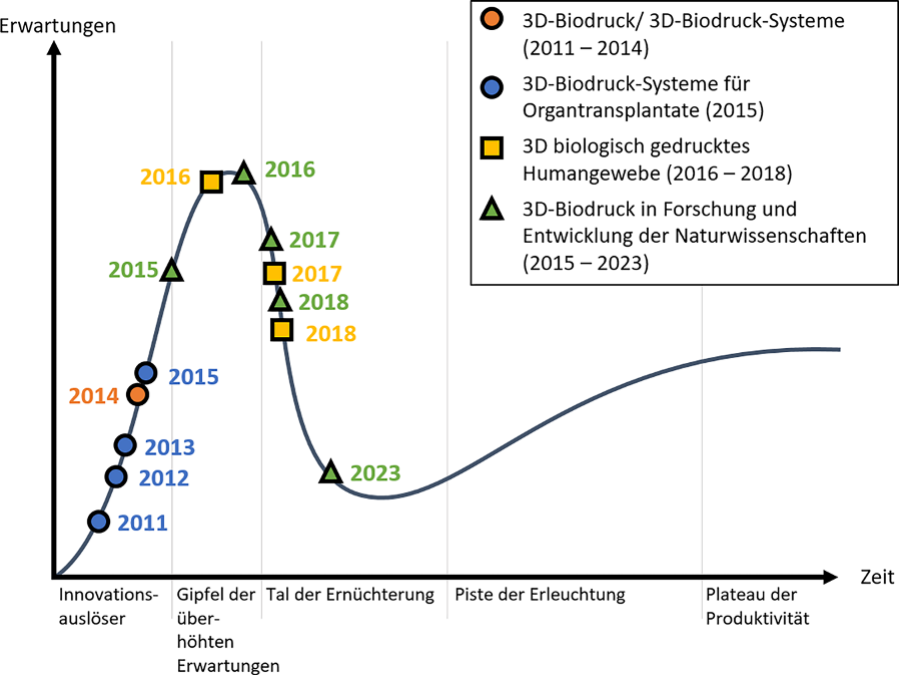
Entwicklung von Begriffen im Zusammenhang mit Bioprinting abgebildet auf dem Gartner Hype Cycle basierend auf den Gartner-Hype-Cycle-for-Emerging-Technologies-Berichten von 2011 bis 2023. Bioprinting war platziert auf dem Höhepunkt der Erwartungen um das Jahr 2016. (Mod. nach [39], mit freundlicher Genehmigung von Elsevier)

Eine unzureichende Analyse und Interpretation bisheriger Ergebnisse im Bereich des 3‑D-Biodrucks kann zu übermäßigen Erwartungen führen, die letztlich nicht praktisch umsetzbar und anwendbar sind (Abb. 1). Der Enthusiasmus für medizinische Forschung ist vor allem aus ethischer Perspektive kritisch zu betrachten, da er die Entscheidungsfindung der Ärzt*innen und ihrer Patient*innen hinsichtlich ihrer Behandlung und der Teilnahme an klinischen Studien beeinflussen könnte [14]. Um den Risiken unrealistischer Prognosen zu begegnen, halten wir es für wesentlich, dass Forschende im Bereich des biologischen 3‑D-Drucks eine objektive Bewertungsmethode wie die Technologiereifegradskala („technology readiness level“, TRL) anwenden, um den Reifegrad ihrer Innovationen objektiv einzuschätzen (Infobox 1: Definition 2). Wir haben hierfür, basierend auf Erkenntnissen gewonnen aus Vorarbeiten der National Aeronautics and Space Administration (NASA) und der United States(US)-Armee [15], wissenschaftlichen Publikationen [16–21], eigenen Projekten [22–27], Vorgaben von Projektträgern [28–30], (etablierten) Marktprodukten [31–33] und Literatur zu Gesetzgebungen und Zulassungsbehörden [27, 34–38], einen Bewertungsrahmen erarbeitet, der es ermöglicht, den Reifegrad einzelner Projekte im Bereich des biologischen 3‑D-Drucks fundiert zu beurteilen. Nach der Beschreibung des Rahmens verwenden wir ihn als Instrument, um relevante systembedingte Barrieren im Bereich biologischer 3‑D-Druckverfahren aufzuzeigen, und wir schlagen vor, wie der Status quo verbessert werden könnte, um die Translation in die klinische Anwendung zu erhöhen.

## Einordnung des biologischen 3-D-Druckverfahrens in den TRL-Rahmen

Das von der NASA entwickelte TRL-System hat sich in zahlreichen Branchen als Standard zur Bewertung des technologischen Reifegrads etabliert, vor allem im Hinblick auf die Marktreife von Innovationen. Die Marktreifephase einer Technologie auf der 9‑stufigen TRL-Skala wurde von O’Connell et al. [39] anhand einer Reihe indikativer Meilensteine bewertet („NASA-Definition“ [39]). Der Aufstieg durch die TRL-Stufen kann mit Bewertungswendepunkten verbunden sein, die insbesondere auch die Zuführung von Investitionsmitteln auslösen [28].

In Deutschland und Europa wird die TRL-Skala oft bei der Vergabe öffentlicher Fördergelder genutzt, um Grundlagen- von angewandter Forschung und Entwicklung abzugrenzen und die Förderquote variiert in Abhängigkeit der Marktreife. Auch im Zusammenhang mit dem biologischen 3‑D-Druckverfahren könnte die TRL-Skala einen robusten und objektiven Maßstab für die Bewertung des Reifegrads von Innovationen und Technologien bieten. So wurde von Naveau et al. [40] bereits eine TRL-Skala für das biologische 3‑D-Druckverfahren vorgeschlagen, jedoch enthielt diese Skala nur eine kurze und, unseres Erachtens, unzureichend ausgearbeitete Skizze der 9 Stufen und nicht die Details, die für eine rationale Bewertung der technologischen Reife erforderlich sind. Dies motiviert die Formulierung einer für TE&RM-Gemeinschaft speziellen biologischen 3‑D-Druckverfahren-spezifischen TRL-Skala in der Veröffentlichung von O’Connell et al. ([39]; „adaptierte TRL-Skala“ [39]). Die dort vorgeschlagene TRL-Skala basiert auf der biomedizinischen TRL-Skala des Medical Research and Material Command (MRMC) der US-Armee, wie sie im „Technology Readiness Assessment“ (TRA-)Deskbook des US-Verteidigungsministeriums enthalten ist [15]. Die TRL-Skalen sind nicht präskriptiv; vielmehr stellen die beschriebenen Richtlinien einen Vorschlag für eine Art Blaupause für die Anforderungen dar, die es zu erfüllen gilt, um ein medizinisches Produkt von der Konzeption zur klinischen Umsetzung zu bringen.

## Bewertung des 3-D-Biodrucks mittels TRL-Skala

Biologische 3‑D-Druckverfahren haben insbesondere das Potenzial, im Bereich chirurgischer Patientenversorgung durch Ansätze aus der regenerativen translationalen Forschung fortschrittliche Lösungen für die Organknappheit zu bieten [41, 42]. Das biologische 3‑D-Druckverfahren befindet sich zwar noch in einem frühen Entwicklungsstadium, hat jedoch bereits seine Einsetzbarkeit zur präzisen Reproduktion von Gewebestrukturen unter Beweis gestellt, was auf dessen potenziell relevanten Stellenwert in der zukünftigen (rekonstruktiven) Chirurgie hinweist [23, 43, 44]. Insbesondere das sog. „point-of-care-bioprinting“ wird in der Chirurgie zunehmend an Bedeutung gewinnen, da es die potenzielle Möglichkeit bietet, patientenspezifische Gewebe und Implantate direkt vor Ort herzustellen, was die Behandlungszeit erheblich verkürzt und die Personalisierung chirurgischer Eingriffe verbessert [1]. Im Jahr 2023 waren weltweit mehr als 100 biologische 3‑D-Druckverfahren-Unternehmen tätig [9]. Der Sektor wächst zwar, aber der Großteil der Branche ist auf die Grundlagenforschung ausgerichtet [41]. Erst kürzlich hat etwa die Bundesagentur für Sprunginnovationen („SPRIND“) entschieden, vier Forschungsteams mit jeweils bis zu einer halben Million Euro zu fördern, um die Produktion künstlicher Organe anzuschieben (Stichwort „Funke – Tissue Engineering“; [45]). Zurzeit gibt es viele kommerziell erhältliche Biotinten und Bioprinter, aber nur sehr wenige Produkte, die biologische 3‑D-Druckverfahren in den Herstellungsschritten verwenden, sind auf dem Markt [41]. Die ersten Produkte, bei deren Herstellung ein Bioprinter eingesetzt wurde, waren das menschliche Lebergewebe „Ex Vivo“ und das menschliche Nierengewebe von Organovo, die 2014 bzw. 2016 auf den Markt kamen. Diese Produkte wurden in nur einer Handvoll veröffentlichter Studien verwendet, die alle von Mitarbeiter*innen von Organovo verfasst oder mitverfasst wurden [16, 17]. Interessanterweise erklärte die Geschäftsführung von Organovo 2018 in einem Interview, „dass ihr bestehendes Geschäft niemals profitabel sein wird und lediglich ein ‚Finanzierungsmechanismus‘ für die Weiterentwicklung ihres Forschungsprogramms ist“ und „aufgrund der großen Diskrepanz zwischen der Größe von Mäuselebern und menschlichen Lebern kommen wir zu dem Schluss, dass NovoTissue bei der Behandlung menschlicher Lebererkrankungen unwirksam sein wird“ (eigene Übersetzung; [46]).

Im Jahr 2022 wurde der erste klinische Fall vorgestellt, bei dem das biologische 3‑D-Druckverfahren bei der Herstellung eines Implantats zur Behandlung einer Ohrmuschel bei einem menschlichen Patienten im Rahmen einer klinischen Studie der Phase 1/Phase 2A eingesetzt wurde [18]. Bei dem in der Entwicklung befindlichen Produkt handelt es sich um AuriNovo, ein patientenindividuelles biologisches Konstrukt zur chirurgischen Rekonstruktion der Ohrmuschel bei Menschen mit Mikrotie Grad II–IV, das von 3‑DBio Therapeutics entwickelt wird [19]. Um diesen wichtigen Schritt zu machen, hat 3‑DBio nach eigenen Angaben eine Biotinte in therapeutischer Qualität (ColVivo) entwickelt, die unter Anforderungen guter Herstellungspraxis („current good manufacturing practice“, cGMP) für den therapeutischen Einsatz verarbeitet wird, sowie einen GMP-kompatiblen 3‑D-Bioprinter genutzt. Diese Studie ist zwar inzwischen vorzeitig terminiert worden (wohl nicht aufgrund von Komplikationen [19]), nichtsdestotrotz ist dies ein wichtiger Meilenstein: Translatorische Barrieren sind überwindbar, sodass durchaus grünes Licht durch Aufsichtsbehörden für die Erstanwendung von Implantaten hergestellt durch biologische 3‑D-Druckverfahren beim Menschen erzielt werden. Jedoch führt dieses einzelne Beispiel nicht dazu, biologischen 3‑D-Druckverfahren generell einen ausgereiften Status zuzuschreiben.

Insbesondere wird TRL nicht auf ein Fachgebiet in seiner Gesamtheit angewandt, z. B. auch bezogen auf Bioprinting, sondern auf einzelne kommerziell orientierte Projekte. In der akademischen Literatur handelt es sich bei der Mehrzahl der veröffentlichten Studien zu biologischen 3‑D-Druckverfahren um Labor- und In-vitro-Charakterisierungsstudien bei TRL 1 bis TRL 3. Nur wenige Studien weisen die Validierungsmerkmale von TRL 4 auf, einer Stufe, die eine unabdingbare Voraussetzung dafür ist, dass die Kompatibilität von Laborprototypen und Datensätzen mit den Standards der Industrieumgebung festgestellt werden kann.

## Warum die meisten Forschungsprojekte zu biologischen 3-D-Druckverfahren einen niedrigen TRL-Wert haben

Aus der Industrieperspektive ist die biologische 3‑D-Druck-Technologie, obwohl schon seit mehr als 30 Jahren erforscht, immer noch in der frühen Innovationsphase, in der es insbesondere um konzeptionelle Neuerungen und die Verbreitung von Technologien geht [47]. Eine solche Phase steht im Einklang mit traditionellen akademischen Publikationsmetriken wie Impact-Faktoren und H‑Indizes, die eher die Publikationsergebnisse als die Auswirkungen auf die reale Welt, also die tatsächliche Umsetzung, bedienen [23]. In einem solchen System gibt es für Forschende Anreize, sich ständig mit der Entwicklung von Technologien zu beschäftigen, viele Projekte parallel durchzuführen, sich ständig um Fördermittel zu bemühen und möglicherweise die potenziellen Auswirkungen der Technologie auf die reale Welt überspitzt abzubilden [48]. Dies schafft auch Anreize für die schnelle, aber oberflächliche Demonstration neuer Konzepte, mit oftmals eingeschränktem Fokus auf Reproduzierbarkeit und Zuverlässigkeit [49] und ohne jemals das Produkt vollständig entlang der TRL-Skala weiter zu entwickeln. Ferner ist translationale Forschung nur eingeschränkt mit den traditionellen wissenschaftlichen Maßstäben vereinbar und behindert gegebenenfalls sogar den Publikationsoutput von Forschenden, insbesondere da diese in der Regel geistiges Eigentum hervorbringt, das vor der öffentlichen Verbreitung angemessen geschützt werden muss. Daher kann sich sogar die Entscheidung zur Durchführung translationaler Forschung negativ auf die akademische Laufbahn in der Wissenschaft tätiger Personen und deren Beschäftigungsaussichten an einer Universität auswirken [49].

## Die translationale Forschung hat einen anderen Charakter als die Frühphase der Innovation

Der Fokus translationaler Forschung, nach unserem Ermessen, ist das Fazilitieren der innovativen Technologie durch die komplette Pipeline mit allen Entwicklungsphasen. Dies erfordert eine langfristige Sichtweise, die alle notwendigen Entwicklungsphasen ernsthaft in Betracht zieht, potenzielle Fehlermöglichkeiten bewertet und den Weg steuert [22]. Forschung, die auf der TRL-Skala weiter fortgeschritten ist, bietet nicht unbedingt ein hohes Maß an konzeptioneller Neuheit, da über die bahnbrechenden Untersuchungen der Funktionsprinzipien bereits berichtet wurde. Oftmals ist es nur der kontinuierlichen Optimierung und der Konzentration auf die Reproduzierbarkeit bekannter/realisierbarer Strategien zu verdanken, dass eine Technologie eine spürbare Wirkung erzielt. Um von der erfolgreichen Translation medizinischer Geräte und insbesondere der Implantatforschung zu lernen, ist es, nach unserem Ermessen, zielführend, wenn die akademische Gemeinschaft im biologischen 3‑D-Druckverfahren über die bloße Technologiedemonstration hinausgeht und sich den strengen Anforderungen stellt, die für die Weiterentwicklung einer Technologie durch die höheren TRLs und schließlich für die Kommerzialisierung gelten.

## Der Weg zur erfolgreichen Translation in die klinische Praxis

Sowohl in Übersichtsartikeln als auch in Ausblickartikeln (sog. „perspectives“) werden häufig die technischen Herausforderungen des Fachgebiets und einige potenzielle Wege zu ihrer Überwindung beschrieben [20, 21]. In Podiumsdiskussionen der TE&RM-Wissenschaftsgesellschaften (z. B. International Society for Biofabrication, Tissue Engineering and Regenerative Medicine International Society und Society for Biomaterials) sowie von Seiten der Industrie und Investoren erhalten die in der akademischen Literatur dargestellten translationalen Herausforderungen jedoch oft nur geringe Aufmerksamkeit [21, 39]. Darüber hinaus lesen sich wissenschaftliche Artikel teilweise mehr wie „Science Fiction Romane“. Ein typisches Beispiel: In einer im Oktober 2020 in *Nature Review Materials* erschienenen Übersichtsarbeit mit dem Titel „3-D-gedruckte multifunktionale Materialien mit Hilfe von Technologien der künstlichen Intelligenz“; Titel im englischen Original „3D-printed multifunctional materials enabled by artificial-intelligence assisted fabrication technologies“ steht in der Konklusion und dem Ausblick des Artikels „Wenn jemand bei einem Autounfall verletzt wird, muss er auf den Rettungswagen warten, der ihn dann ins Krankenhaus transportiert, wodurch sich die kritische Versorgung verzögert. Unsere Vision ist, dass in Zukunft jeder einen 3‑D-Drucker im Kofferraum seines Autos hat, mit dem er am Unfallort direkt ein biomedizinisches Gerät für den Patienten am Unfallort drucken kann. Die Entwicklung von 3‑D-druckbaren elektrischen und biologischen Materialien hat die Möglichkeiten der additiven Fertigungstechnologien erweitert und ermöglicht nun die Herstellung von maßgeschneiderter Kleidung mit medizinischer Sensorik und Implantaten mit verschiedenen Funktionen, wie zum Beispiel integrierte Rechenleistung, Gesundheitsüberwachung und Gewebereparatur“ (eigene Übersetzung; [50]).

**Abb. 2:**
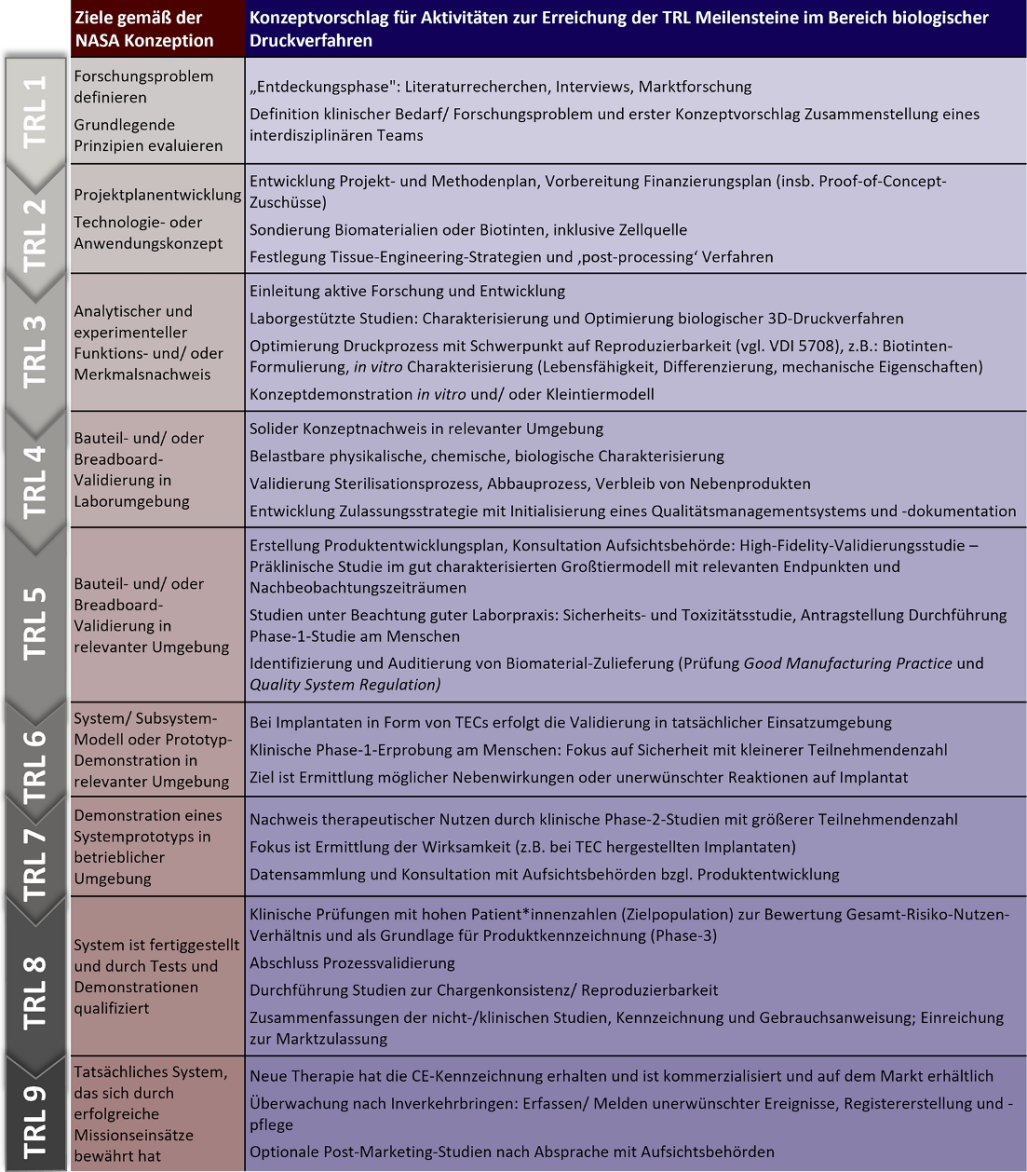
TRL-Ziele gemäß der ursprünglichen NASA-Konzeption und entsprechender Konzeptvorschlag für dessen Anwendung im Bereich biologischer Druckverfahren für implantierbare 3‑D-gedruckte Konstrukte. (Abb. in Anlehnung an [39], mit freundlicher Genehmigung von Elsevier.) *CE* Conformité Européenne, *TEC* „tissue engineered construct“

Unabhängig davon, ob die zahlreichen technischen Herausforderungen gemeistert werden, klafft zwischen dem aktuellen „Stand der Technik“ und dem, was erforderlich ist, um eine biologische 3‑D-Druckverfahren-Technologieplattform vom Labor in die klinische Praxis zu übertragen, derzeit noch eine signifikante Lücke (sog. Translationslücke; [51]). Die Erforschung, Entwicklung und Umsetzung medizinischer Produkte wie Implantate aus Gewebe ist ein komplexer und mehrstufiger Prozess, weshalb wir für die jeweiligen TRL-Skalen Handlungsempfehlungen aufzeigen (Abb. 2). Hier präsentieren wir einige Denkanstöße, die auf einem Zitat von Konfuzius basieren: „Lernen ohne Denken ist verlorene Arbeit; Denken ohne Lernen ist gefährlich“, für deren Beachtung durch die biologische 3‑D-Druckverfahren-Gemeinschaft in den Bereichen akademische Veröffentlichung, Übersetzung und Kommerzialisierung wir werben. In diesem Zusammenhang heben wir nun einige der systembedingten Barrieren hervor, die wir vorschlagen zu adressieren, um die Umsetzung biologischer 3‑D-Druckverfahren-Technologien auf breiter Ebene zu unterstützen.

### Verstärkte Betonung der Reproduzierbarkeit

TRL 4 erfordert die Durchführung „robuster“ Validierungsstudien. Um diese Stufe zu erreichen, ist eine strenge Anwendung quantitativer Methoden erforderlich, einschließlich statistischer Analysen an repräsentativen Datensätzen und der Messung des sog. „Best-practice“-Beispiels, das die beste Leistung aufweist. Leider fehlt in der Literatur zu biologischen 3‑D-Druckverfahren derzeit ein Schwerpunkt auf der Reproduzierbarkeit. Die Anzahl der für biologische Tests vorbereiteten Proben in Publikationen zu biologischen 3‑D-Druckverfahren liegt in der Regel bei *n* = 3, selbst bei den am häufigsten zitierten Publikationen in den Zeitschriften mit dem höchsten Impact [52]. Inzwischen veröffentlichen einige angesehene Zeitschriften Arbeiten, die auf nur zwei [53] oder sogar nur einer 3‑D-gedruckten Probe basieren [54]. Es scheint, die Forschungsgemeinschaft im Bereich biologische 3‑D-Druckverfahren ist insbesondere an der Demonstration immer höherer Komplexität interessiert und nicht an Reproduzierbarkeit und Skalierung.

### Flächendeckende Adhärenz zur Definition von Standards für Versuchsmethoden und ihre breite Einführung

Experimentelle Protokolle und standardisierte Arbeitsanweisungen („standard of procedure“, SOP) gewährleisten Reproduzierbarkeit. Außerdem erleichtern klare Protokolle die Kommunikation und Zusammenarbeit innerhalb der wissenschaftlichen Gemeinschaft und sind von großem Nutzen bei der Erstellung systematischer Übersichten und Metaanalysen. Regulierungsbehörden können vorschreiben, dass Prozesse, wie z. B. in der Qualitätskontrolle, Sterilisation, Biokompatibilitätstests, nach Standardmethoden durchgeführt werden, wie sie von internationalen Instituten wie American Society for Testing Materials (ASTM) und International Organization for Standardization (ISO) beschrieben sind, und tun dies auch. Unseres Wissens wurden bisher jedoch nur zwei biologische 3‑D-Druckverfahren-spezifische Normen als Entwürfe vorgelegt, die sich beide auf die Druckbarkeit extrusionsbasierter Biotinten beziehen, nämlich ASTM WK65680: „New Test Methods for Printability of Bioinks and Biomaterial Inks“ und WK72274: „New Test Method for Printability of Bioinks and Extrusion-Based Bioprinting“. Das Fehlen geltender Standards im Bereich der biologischen 3‑D-Druckverfahren ist eine Barriere für die Umsetzung und muss durch eine stärkere Direktive der internationalen TE&RM-Fachgesellschaften, die viele Forschenden und Anwendenden des biologischen 3‑D-Druckverfahrens als Mitglieder haben, behoben werden. In jüngster Zeit sind Konsortien entstanden, wie z. B. die Regenerative Medicine Manufacturing Society [55] und das Advanced Regenerative Manufacturing Institute [56], die sich dazu verschrieben haben, dieses Problem zu adressieren, obwohl diese Organisationen jedoch noch keine neuen Standards veröffentlicht haben. Positiv hervorzuheben ist die Arbeit des deutschen Richtlinienausschusses VDI 5708, der eine technische Regelsetzung für das Bioprinting erarbeitet hat, die seit Dezember 2023 als Entwurf einsehbar ist [27].

### Berücksichtigung und Anwendung der regulatorischen Anforderungen

Ziel einer Zulassungsstrategie ist es, den Weg zur Erfüllung der Anforderungen von Zulassungsbehörden wie der U.S. Food and Drug Administration (FDA), der European Medicines Agency (EMA) oder der Therapeutic Goods Administration (TGA) in Australien zu skizzieren [34]. Nach der aktuellen Gesetzgebung werden zellbasierte Arzneimittel, bei denen bearbeitete Zellen mit einem Materialgerüst kombiniert werden, welches das Wachstum neuen Gewebes physikalisch unterstützt, in den United States of America (USA) als Biologika und in der Europäischen Union (EU) als kombinierte Advanced Therapy Medicinal Products (ATMPs) reguliert [35]. In dem vorliegenden Beitrag liegt der Fokus auf Europa und biologischem 3‑D-Druck (Bioprinting).

Die nationalen und zentralisierten Bestimmungen bilden zusätzlich zu den relevanten Richtlinien für Medizinprodukte den Regulierungsrahmen, der alle kritischen Aspekte der Entwicklung, Herstellung, Zulassung und anschließenden Überwachung nach dem Inverkehrbringen für Kombinations-ATMPs steuert. Dazu gehören regulatorische Beiträge zur Festlegung der Qualitäts- und Sicherheitsstandards für die Spende, Beschaffung und Prüfung menschlicher Zellen oder Gewebe gemäß der EU-Gewebe- und Zellrichtlinie. Dazu gehören auch Standards für die Herstellung von Produkten unter Einhaltung der GMP-Grundsätze und für die Durchführung klinischer Studien in der EU gemäß den Grundsätzen für gute klinische Praxis („good clinical practice“, GCP; [35]).

Da die regulatorischen Erwägungen einen großen Einfluss auf die Translationsaktivitäten nach dem Konzeptnachweis haben, sollte die regulatorische Strategie früh im Produktentwicklungsprozess entwickelt und während des gesamten Lebenszyklus des Produkts bei Bedarf aktualisiert werden. Die Entwicklung einer regulatorischen Strategie ist eine Aktivität bei TRL 4 in unserem vorgeschlagenen TRL-Rahmen.

Die ganzheitliche Betrachtung von Zellen, Materialien (nicht nur Biotinten, sondern alle im 3‑D-Drucker und im Druckprozess verwendeten Materialien) und Verarbeitungsbedingungen, die für das biologische 3‑D-Druckverfahren erforderlich sind – einschließlich Art und Reinheit – und die für die Verwendung am Menschen zugelassen sind, ist in der Forschung zu biologischen 3‑D-Druckverfahren nicht in dem gebotenen Umfang und der notwendigen Tiefe vorhanden. Insbesondere im Bereich des akademischen Forschungssektors ist, nach unserer Einschätzung, Potenzial zu unterstützen, das Verständnis hinsichtlich der Komplexität notwendiger regulatorischer Prozesse zu vermitteln [36]. In Ermangelung einer Prädikatstherapie sind die regulatorischen Anforderungen an biologische 3‑D-Druckverfahren-Technologien jedoch oftmals nicht eindeutig [34, 57]. Tatsächlich ist die regulatorische Landschaft in gewisser Weise im Fluss, wobei verschiedene Konsultationen und Stellungnahmen von globalen Regulierungsbehörden hinsichtlich der zukünftigen Klassifizierung und des regulatorischen Prozesses im Zusammenhang mit biologischen 3‑D-Druckverfahren verfasst wurden [58].

### Durchführung der Forschung im Rahmen eines Qualitätsmanagementsystems

Qualitätsmanagementsysteme (QMS) sind in der Gesundheitsindustrie von entscheidender Bedeutung, um die Qualität von Therapien und die Minimierung von Risiken zu gewährleisten. Ein QMS ist ein strukturiertes System von Prozessen, die alle Aspekte unter anderem der Entwicklung, der Herstellung, des Lieferantenmanagements, des Risikomanagements, der klinischen Daten, der Lagerung, des Vertriebs und der Produktkennzeichnung abdecken. Die meisten wichtigen Märkte verlangen die Einführung und Aufrechterhaltung eines QMS als Bedingung für die Produktregistrierung [34]. So orientieren sich beispielsweise die Herstellerfirmen von kombinierten ATMPs in Europa in der Regel an der Norm ISO 13485, während die US-amerikanischen Unternehmen die Quality System Regulation (QSR) der U.S. Food and Drug Organisation (FDA) einhalten. Die Komplexität des QMS hängt von der Klassifizierung des Produkts ab, wobei für Hochrisikoprodukte, wie z. B. implantiertes durch biologischen 3‑D-Druck hergestelltes künstliches Gewebe, die strengsten Anforderungen gelten. Wie in der obigen TRL-Skala dargestellt, wird das anfängliche QMS-System in der Regel bei TRL 4 eingerichtet und im weiteren Verlauf der Umsetzungsphase weiterentwickelt.

### Nutzung von GMP-Laboren und zertifizierten Instituten

„Good manufacturing practice“ ist ein weiteres System von Prozessen, die sicherstellen, dass Produkte reproduzierbar hergestellt und gemäß den für ihren Verwendungszweck geltenden Qualitätsstandards kontrolliert werden. Während das QMS alle Prozesse innerhalb einer Organisation umfasst, die sich auf die Produktqualität auswirken könnten, konzentriert sich die GMP auf die Herstellung. Einrichtungen, die unter GMP arbeiten, sind in der Pipeline bis zur klinischen Umsetzung unerlässlich, um die Qualität, Sicherheit und Wirksamkeit therapeutischer Produkte zu gewährleisten. GMP-Einrichtungen spielen vor allem bei der Herstellung von Zelltherapieprodukten [59], wie z. B. bei künftigen biologisch 3‑D-gedruckten Produkten, eine entscheidende Rolle. In der EU ist beispielsweise eine Herstellungserlaubnis für alle Phasen der Entwicklung klinischer Versuche erforderlich, und diese Erlaubnis ist häufig an die Herstellung der Therapie in einer GMP-Einrichtung gebunden (siehe EudraLex Band 4; [60]).

### Verwendung zertifizierter Biomaterialien/medizinischer Werkstoffe

Medizinische Werkstoffe sind solche, die für den Einsatz in Produkten für humane Endanwender geeignet sind und oft ein sog. FDA „Material Master File“ haben. Solche Materialien müssen die ISO 10993 erfüllen, in der die Anforderungen an die Biokompatibilität festgelegt sind. Es ist wichtig, bereits ab den frühen TRL-2–4-Phasen der Entwicklungspipeline konsistent die Werkstoffe in der endgültigen Materialformulierung zu verwenden, da für die Zulassung für künftige Therapien eine Werkstoffkonsistenz während der gesamten Validierungsphase nachzuweisen ist.

### Buisinessplan (Geschäftsentwicklungsplan)

Eine Marktzulassung reicht nicht aus, um eine wirkungsvolle medizinische Therapie zu entwickeln, bei der sog. „tissue engineered constructs“ (TECs) als Implantate verwendet werden, deren Herstellung mittels biologischer 3‑D-Druckverfahren erfolgt. Zum Beispiel muss das Produkt unter sterilen Bedingungen hergestellt und dann verpackt werden. Ein Geschäftsentwicklungsplan für eine neue medizinische Therapie ist ein umfassendes Dokument, das die Strategie für die Entwicklung, Kommerzialisierung und Marktdurchdringung der Therapie umreißt und Einzelheiten zur Produktentwicklung, zur Zulassungsstrategie, zur Marktanalyse, zur Marketing- und Vertriebsstrategie, zu den Betriebsabläufen, zu den finanziellen Prognosen und zum Risikomanagement enthält.

Wichtig ist, dass der Geschäftsplan nicht erst *nach* der Entwicklung der Technologie erstellt werden sollte. Vielmehr wird der Geschäftsplan in anderen Branchen in der Regel parallel zur Technologie entwickelt. Idealerweise beginnt der Businessplan 1.0 bei TRL 1, also in der Phase der Marktforschung. Externe Investorinnen und Investoren können einen detaillierten Geschäftsplan als Bedingung für ihre Investition in jeder Phase der Entwicklungspipeline verlangen.

### Produktion und Post-Market Surveillance

Wir haben bereits einige Qualitätskontrollprozesse erwähnt, die erforderlich sind, um die TRL-Stufen zu durchlaufen. Die Markteinführung und der sog. „scale-up“ nach der Marktzulassung stellen zusätzliche Herausforderungen dar. Das Herstellungsverfahren muss in der Lage sein, das Produkt in größeren Mengen zu produzieren, ohne dass die Qualität darunter leidet. Die Herstellungsprozesse müssen kosteneffizient sein, um die kommerzielle Lebensfähigkeit des Produkts zu gewährleisten. Klinikerinnen und Kliniker sollten rekrutiert und geschult werden, und es müssen Compliance-Tests durchgeführt werden, um sicherzustellen, dass das Produkt nach validierten Verfahren verwendet wird.

## Fazit

Biologische 3‑D-Druckverfahren sind seit mehr als zwei Jahrzehnten Gegenstand zunehmender Forschung und Innovation, jedoch haben sich die hohen Erwartungen an eine zeitnahe Umsetzung dieser Technologien in die (routinemäßige) klinische Patientenversorgung bisher nicht erfüllt. Trotz bedeutender Fortschritte bleiben die Entwicklungen oft im akademischen Bereich und Forschungsumfeld verankert. Dies hat eine Dynamik geschaffen, in der die wahrgenommenen Vorteile des 3‑D-Bioprintings primär im wissenschaftlichen Kontext zum Tragen kommen, während die direkte Integration in die klinische Praxis, insbesondere zur Verbesserung der Patientenversorgung, nur schleppend voranschreitet. Traditionell steht die biomedizinische Forschung vor der Herausforderung, sowohl die Grundlagenforschung als auch die Entwicklung marktreifer Produkte voranzutreiben. Um die tatsächlichen Vorteile des 3‑D-Bioprintings für die Patientenversorgung voll auszuschöpfen, ist ein stärkerer Fokus auf die praktische Anwendbarkeit, die regulatorischen Anforderungen, die Skalierbarkeit in der Produktion sowie den kommerziellen Erfolg notwendig. Nur durch eine enge Verzahnung dieser Aspekte kann die Translation vom Labor in die klinische Routine und die Verbesserung der chirurgischen Patientenversorgung erfolgreich gelingen.

### Infobox Definitionen

*Definition 1: Endprodukt nach biologischen 3‑D-Druckverfahren*

Die Definition des Endprodukts nach biologischen 3‑D-Druckverfahren ist komplex, da es sich um ein Medizinprodukt oder ein Zubehör zu einem Medizinprodukt handeln könnte, das unter die Richtlinie 93/42 fällt [37]: Das Produkt kann entsprechend ein Arzneimittel für neuartige Therapien („advanced therapy medicinal product“, ATMP) sein, das unter die Verordnung 1394/2007 fällt, oder sogar ein Arzneimittel, das unter die Richtlinie 2001/83 fällt. Bei dem Rohmaterial kann es sich um chemische Stoffe handeln, die unter die Verordnung 1907/2006 (REACH) fallen, oder um lebende Zellen und Gewebe, die unter die Richtlinie 2004/23 fallen [37].

Die Kombination eines 3‑D-gedruckten Medizinprodukts mit einem Arzneimittel bringt eigene regulatorische Herausforderungen mit sich: Die Regulation hängt davon ab, ob die therapeutische Wirkung für den Patienten hauptsächlich über das Arzneimittel erfolgt oder hauptsächlich auf das Medizinprodukt zurückzuführen ist [38].

*Definition 2:* *„Technology readiness level“ (TRL)*

Der „technology readiness level“ (TRL), auf Deutsch als Technologiereifegrad übersetzt, ist eine Skala zur Bewertung der Markreife neuer Technologien auf der Basis einer systematischen Analyse. Er gibt auf einer Skala von 1 bis 9 an, wie weit eine Technologie zur Herstellung eines Produktes entwickelt worden ist. Entwickelt wurde die TRL-Skala 1988 von der NASA für die Bewertung von Raumfahrttechnologien, davon ausgehend hat sie sich als Standard in weiteren Bereichen wie z. B. Automobil, Luftfahrt und insbesondere der Zukunftstechnologien etabliert.
